# Influence of Obesity on Foot Loading Characteristics in Gait for Children Aged 1 to 12 Years

**DOI:** 10.1371/journal.pone.0149924

**Published:** 2016-02-25

**Authors:** Steffen Mueller, Anja Carlsohn, Juliane Mueller, Heiner Baur, Frank Mayer

**Affiliations:** 1 University Outpatient Clinic, Sports Medicine & Sports Orthopaedics, University of Potsdam, Potsdam, Germany; 2 University of Education Schwäbisch Gmünd, Institute of Health Sciences, Schwäbisch Gmünd, Germany; 3 Bern University of Applied Sciences, Health, Physiotherapy, Bern, Switzerland; West Virginia University, UNITED STATES

## Abstract

**Background:**

Overweight and obesity are increasing health problems that are not restricted to adults only. Childhood obesity is associated with metabolic, psychological and musculoskeletal comorbidities. However, knowledge about the effect of obesity on the foot function across maturation is lacking. Decreased foot function with disproportional loading characteristics is expected for obese children. The aim of this study was to examine foot loading characteristics during gait of normal-weight, overweight and obese children aged 1–12 years.

**Methods:**

A total of 10382 children aged one to twelve years were enrolled in the study. Finally, 7575 children (m/f: n = 3630/3945; 7.0±2.9yr; 1.23±0.19m; 26.6±10.6kg; BMI: 17.1±2.4kg/m^2^) were included for (complete case) data analysis. Children were categorized to normal-weight (≥3^rd^ and <90^th^ percentile; n = 6458), overweight (≥90^rd^ and <97^th^ percentile; n = 746) or obese (>97^th^ percentile; n = 371) according to the German reference system that is based on age and gender-specific body mass indices (BMI). Plantar pressure measurements were assessed during gait on an instrumented walkway. Contact area, arch index (AI), peak pressure (PP) and force time integral (FTI) were calculated for the total, fore-, mid- and hindfoot. Data was analyzed descriptively (mean ± SD) followed by ANOVA/Welch-test (according to homogeneity of variances: yes/no) for group differences according to BMI categorization (normal-weight, overweight, obesity) and for each age group 1 to 12yrs (post-hoc Tukey Kramer/Dunnett’s C; α = 0.05).

**Results:**

Mean walking velocity was 0.95 ± 0.25 m/s with no differences between normal-weight, overweight or obese children (p = 0.0841). Results show higher foot contact area, arch index, peak pressure and force time integral in overweight and obese children (p<0.001). Obese children showed the 1.48-fold (1 year-old) to 3.49-fold (10 year-old) midfoot loading (FTI) compared to normal-weight.

**Conclusion:**

Additional body mass leads to higher overall load, with disproportional impact on the midfoot area and longitudinal foot arch showing characteristic foot loading patterns. Already the feet of one and two year old children are significantly affected. Childhood overweight and obesity is not compensated by the musculoskeletal system. To avoid excessive foot loading with potential risk of discomfort or pain in childhood, prevention strategies should be developed and validated for children with a high body mass index and functional changes in the midfoot area. The presented plantar pressure values could additionally serve as reference data to identify suspicious foot loading patterns in children.

## Introduction

Overweight and obesity are increasing health problems that are not restricted to adults only. In the developed countries, the prevalence of childhood overweight and obesity was 7.9% in 1990 and is estimated to be as high as 14.1% in 2020 [1].

There is evidence that childhood obesity is associated with various metabolic and psychological comorbidities [2]. However, only few data is available about the impact of childhood overweight or obesity on the musculoskeletal system. Recently, Yan et al. have shown that childhood obesity may affect plantar pressure distribution and walking stability in obese children compared to non-obese subjects due to the overload of foot structures [3]. The lower extremities and particularly the feet are exposed to additional mass in everyday life. Thus, about 0.5 times of body mass has to be tolerated by the foot during standing. During normal gait, a load of 1.2 body mass must be carried by the foot and increased load of body mass 2–3 fold during running [4–7].

It is well known that obesity in adults is associated with changes in gait pattern and loading of the lower extremities and the foot [8,9]. The midfoot area (longitudinal arch) was affected the most (3 times higher peak plantar pressure) in obese compared to normal-weight male and female adults [8]. Beyond geometric and pressure changes, overweight and obese adults are more likely to suffer from overuse injuries at the lower extremities than normal-weight persons [10]. Mickle & Steele (2015) reported that obese adults suffer from altered foot function and foot pain which directly impact mobility and quality of life of those affected [11]. These studies support the theory that increased stress on the soft tissues and joints, which may be directly related to high body mass, is associated with higher prevalence of foot discomfort, pain and therefore reduced level of physical activity [9,11–13].

In children and adolescents, growth and maturation may additionally influence changes of foot pattern and foot loading caused by overweight. It is known that foot morphology (e.g. relative foot width, foot type: flatfoot) in children is changing during maturation and is different between boys and girls [14–16]. Focusing on foot morphology in relation to obesity, Riddiford-Harland et al. (2000) found differences between normal-weight and obese children with an increased Chippaux-Smirak Index characterizing a reduced longitudinal foot arch for obese children [17]. Therefore, more detailed information on foot function e.g. with plantar pressure measurements as a valid and reliable tool seems reasonable (18;19). Already in static test situations (e.g. during standing) obese children display higher loading on the foot compared to non-obese children [12,18,19].

In walking, higher foot contact area and higher foot loading in obese compared to non-obese children is evident [12,19,20]. Dowling et al. (2004) showed higher foot contact area and higher foot loading, except for the toes, in obese compared to non-obese children 9 years of age [12]. These results are supported by Filippin et al. (2007) analyzing children aged 9 to 11 years [19]. In addition, Cousins et al (2013) demonstrated a foot loading pattern with higher peak pressure and peak force under the midfoot and metatarsal regions (2^nd^ to 5^th^) in obese children between 7 to 11 years of age [20]. Moreover, Riddiford-Harland et al. (2015) reported that already in children aged 8 years increased plantar pressure under the feet in obese and overweight is directly associated to children’s physical activity level [21]. Higher plantar pressure values were also found in prepubescent children during walking with self-chosen walking velocity [3]. The results show highest change in plantar pressure for the midfoot region. In contrast, for children aged 4 to 7 years with normal development, Phethean et al. (2012) did show a low to moderate association between body mass and body mass index with plantar pressure measures with no differences between girls and boys [22]. Therefore, higher plantar pressure values for the total foot are expected for persons with higher body mass independent of age or gender. In contrast, the load distribution across foot regions is expected to be disproportional for different body masses. The authors assume that plantar pressure data do not necessarily have to be normalized to body mass and that data of girls and boys may be pooled for data analysis.

Thus, it could be hypothesized, that overweight may not affect the foot shape or function of small children as much as it does in older children. Nevertheless, there is still a lack of knowledge, if body mass affects plantar pressure of the feet already in children younger than 4 years of age. Mainly caused by small sample sizes or narrow age range, the development of foot loading in obese and overweight children over the course of aging and maturation from 1 to 12 years of age is not detailed.

Therefore, the aim of the study was to examine the effect of different body mass in normal-weight, overweight and obese children aged 1–12 years on plantar pressure distribution characteristics during gait. Moreover, it was aimed to identify characteristic foot loading patterns in comparison of normal-weight, overweight and obese children in all age groups and consideration of different foot regions.

## Methods

### Subjects and study design

The study was performed in 61 cities across Germany with volunteers recruited at information desks located near the measuring stations. Information about the purpose, methods and potential risks were given to the children’s parents. Afterwards, parents gave written informed consent before children’s voluntary participation in the study. The study was approved by the Ethical Commission of the Albert-Ludwigs University Freiburg.

A total of 10382 children aged one to twelve years were enrolled in the study. Children were excluded from the study if they were unable to walk unsupported, were not compliant to complete the entire measurement set-up (n = 161) or suffered from recent injuries or pain during gait (n = 52). These children showed no statistical significant difference in anthropometric data compared to the remaining cohort. Subjects (N = 2557) were excluded from the data analysis in the case of any missing values (i.e. age, height, body mass or less than three repetitions on the walkway) or implausible data. Children were categorized as underweight (<3^rd^ percentile; n = 37), normal-weight (≥3^rd^ and <90^th^ percentile; n = 6458), overweight (≥90^rd^ and <97^th^ percentile; n = 746) or obese (>97^th^ percentile; n = 371) according to the German reference system that is based on age and gender-specific body mass indices (BMI) [23,24]. Children categorized as underweight (n = 37) were excluded for further statistical analysis due to missing subjects in many age groups. Finally, 7575 children (m/f: n = 3630/3945; 7.0±2.9y; 1.23±0.19m; 26.6±10.6kg; BMI: 17.1±2.4kg/m^2^) were included into data analysis. Participants’ anthropometrics are shown in Table 1.

**Table 1:** Anthropometrics.

	n	n (f/m)	Age[years]		Body mass [kg]		Height [m]		BMI [kg*m^-2^]		Foot length [cm]		Foot width [cm]	
			Mean	SD	Mean	SD	Mean	SD	Mean	SD	Mean	SD	Mean	SD
**all**	7575	3945/3630	7.0	2.9	26.6	10.6	1.23	0.19	17.1	2.4	19.1	2.9	7.3	0.9
**female**	3945		7.0	2.8	26.6	10.6	1.23	0.19	17.0	2.4	19.0	2.9	7.2	0.8
**male**	3630		6.9	2.9	26.6	10.6	1.22	0.19	17.1	2.3	19.1	3.0	7.4	0.9
**normal weight**	6458	3384/3074	7.0	2.8	25.4	9.0	1.22	0.18	16.4	1.5	19.0	2.8	7.2	0.8
**over-weight**	746	367/379	6.8	3.1	31.4	13.2	1.23	0.21	19.7	1.9	19.4	3.3	7.6	1.0
**obese**	371	194/177	7.2	3.2	39.0	17.5	1.26	0.23	23.1	3.3	20.0	3.5	7.9	1.1

The measurement protocol always started with the assessment of anthropometric data (age, body height, body mass) with the barefoot subjects wearing their everyday clothes. To avoid additional bias, body weight was not corrected for the weight of clothes by generally subtracting e.g. 1 kilogram of the measured body mass. Foot geometry (foot length and width [cm]) was measured during stance (WMS® foot measurement system, with an attached millimeter scale, DSI, Offenbach, Germany) followed by gait measurements. For standardized plantar pressure measurements of children’s gait a portable instrumented walkway (length: 3.0 m; width 0.8 m, height 4.0 cm) was used [16]. To acquire comfortable natural barefoot walking pattern, the surface of the wooden walkway was covered with 1.0 cm thick tartan comparable to a polyurethane surface of athletic tracks and masked with a thin cloth. Therefore, a targeting of the measurement platform, leading to an artificial walking pattern, was minimized. Walking speed was determined using a photoelectric barrier positioned in the middle of the 3 m walking distance (measurement distance: 1.0 m; measurement height: 54 cm; Timer S4, Alge Timing®, Lustenau, Austria). Plantar pressure distribution and loading patterns during gait were assessed using a pressure measurement platform that was mounted in the walkway (Emed X®, Novel GmbH®, Munich, Germany). The pressure measurement platform consisted of a 40 cm x 69 cm sensor matrix with a resolution of 4 sensors / cm² auto calibrated before each measurement. The sampling rate was determined at 100 Hz and auto triggered at first contact.

For familiarization, children were encouraged to repeatedly walk on the walkway. Afterwards, plantar pressure of the right foot was measured during barefoot gait with self-selected speed across the instrumented walkway. To account for differences in gait variability for predefined walking velocities, walking speed was not standardized in the present study [16,25]. The upper limit of walking velocity was chosen at 6 km/h due to the reported transition of walking to running in adults at this speed [26,27]. A minimum of three and a maximum of five completed attempts were required. To ensure reproducible gait pattern, at least one stride had to be completed before entering the measurement area within the photoelectric barriers. The straightforward two-step protocol was shown to have higher reproducibility for plantar pressure measurements during gait compared to a midgait measurement [28]. All investigations were performed by the same experienced examiner, supported by two additional scientific assistants using a portable and instrumented walkway.

### Measures

Main outcome measures were (1) the contact area (CA [cm²]) to calculate the arch index, (2) the force time integral (FTI [N∙s]) and (3) the peak pressure (PP [kPa]) as representatives for the foot loading. CA, FTI and PP are well accepted, reliable and valid variables to characterize the longitudinal foot arch, the overall and the maximum isolated loading of the foot [19,29,30]. The measures were acquired for the total foot, toes, forefoot, medial midfoot, lateral midfoot and hindfoot using Novel-win software package (Novel Projects® & Novel Scientific®, Munich, Germany). The hindfoot, midfoot and forefoot are defined (by default; Novel Automask) using 27% and 55% of the foot length from heel to toes and medial/lateral midfoot is divided by the foot length axis, respectively. For analysis, the toes were separated from forefoot using pressure gradients around the peak pressures of the toes and were only included for measures of the total foot. Additionally, the dynamic arch index (AI) was calculated as described by Cavanagh et al. (1987)[30]. According to Cavanagh´s definition, a high arch results in a small arch-index, whereas a flat arch leads to a large arch-index. Therefore the AI is used as indirect measure for longitudinal foot arch characteristics. All plantar pressure measurements were calculated for each subject from the mean out of 3 to 5 valid walking trials.

### Data analysis

All data were checked for plausibility with a range check (age: 1–12 years; body size: < 200 cm; body weight: < 120 kg; BMI > 12 kg/m²; gait speed: < 6 km/h). Implausible values were recalculated or corrected as documented in the CRF. Remaining extreme values were excluded. For further analysis 12 age groups (1 to 12 years age) were built based on the subjects’ birth dates.

Data was analyzed descriptively (mean ± SD) followed by ANOVA/Welch-test (according to homogeneity of variances: yes/no) for group differences according to BMI categorization (normal-weight, overweight, obesity) and for each age group 1 to 12yrs (post-hoc Tukey Kramer/Dunnett´s C; global α = 0.05; with Bonferroni adjustment for multiple comparisons in 3 weight and 12 age groups: adjusted α = 0.001) (JMP Statistical Software Package 8.0.1a, SAS Institute®). In addition, the differences of the obese children were given in relation to the normal-weight children (x-fold).

## Results

Mean walking velocity was 0.95 ± 0.25 m/s with no differences between normal-weight, overweight or obese children (p = 0.0841). In general, total foot contact area (CA [cm^2^]) successively rose from the youngest (47.2 ± 5.7 cm^2^) to the oldest children (101.8 ± 17.0 cm^2^). Overall, and in each age group, the normal-weight children presented a smaller CA than overweight children (p<0.001). Highest values of foot contact area were found in obese children.

**Fig 1:**
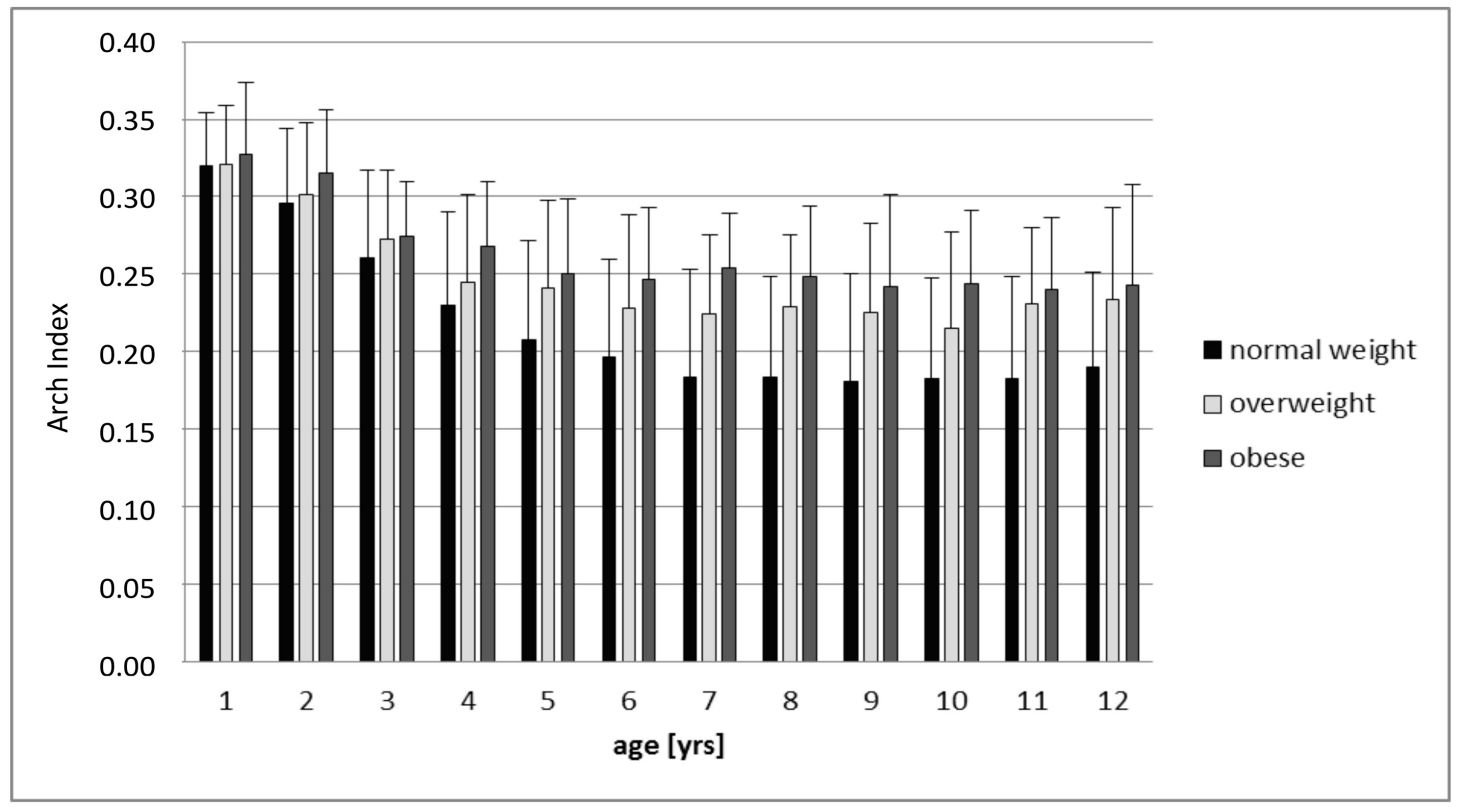
The Arch index (AI) in 1 to 12 year old normal-weight, overweight and obese children with mean ± SD.

The smallest arch index (AI) was seen in normal-weight (0.21±0.07) followed by overweight (0.25±0.06) and obese children (0.26±0.05; p<0.001). In the single age groups (1-12yrs), the normal-weight children presented smaller AI than overweight or obese children (Fig 1). The finding of smaller AI in normal-weight compared to overweight or obese children was statistically significant for the age groups 5 to 12 years (p<0.001), but not for children aged 1 to 4 years (p>0.001).

**Fig 2:**
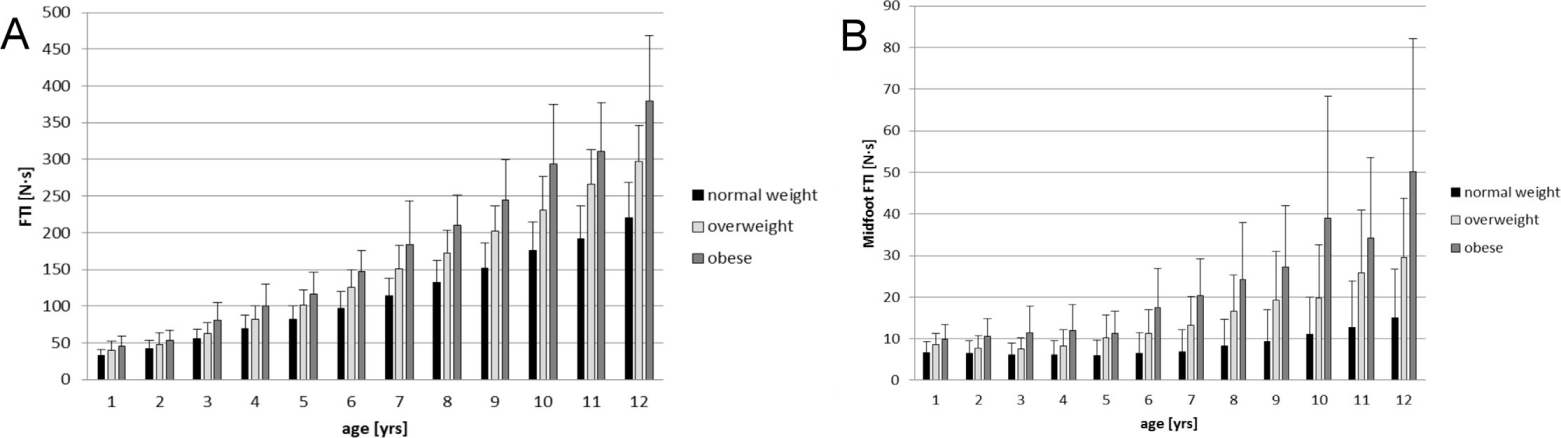
The force time integral (FTI [N∙s]) in 1 to 12 year old normal-weight, overweight and obese children with mean ± SD. A: for the total foot; B: for the midfoot.

A consistent increase of the overall foot loading (quantified by: FTI total foot) from 1 to 12 year-old children was found. In each age group, FTI values of the total foot were highest for the obese and lowest for the normal-weight children. (Fig 2A and Table 2). Obese children displayed the 1.26-fold (2 years-old) up to the 1.75-fold (12 years-old) of the total foot loading (FTI) that was observed in normal-weight children of the same age. The amount of statistical significant group differences was especially present for the total foot and forefoot starting at the age of 3-4years. For the rearfoot and midfoot area differences are visible from an age of 5-6years (Table 2; p<0.001).

**Table 2:** The force time integral (FTI [N∙s]) for the foot region: toes, forefoot (FF), medial midfoot (MMF), lateral midfoot (LMF), rearfoot (RF), total foot (TF) with mean ± SD.

age [yrs]	normal weight (1); overweight (2); obese (3)	n	Toes		FF		MMF		LMF		RF		TF	
			Mean	SD	Mean	SD	Mean	SD	Mean	SD	Mean	SD	Mean	SD
**1**	1	108	5.0	2.3	14.1	5.8	2.6	1.7	4.1	1.8	7.6	3.4	32.8	7.8
	2	29	5.6	4.3	17.2	7.2	3.4	2.2	5.1	1.9	8.8	5.1	39.8	12.8
	3	20	5.8	3.2	18.8	9.6	3.5	1.6	6.5	2.8	10.8	5.2	45.1	14.0
**2**	1	348	6.7	3.3	17.3	6.4	2.4	1.6	4.1	2.2	12.0	5.7	42.2	11.8
	2	69	6.7	3.2	20.6	10.9	2.7	1.5	5.0	2.0	13.2	6.8	47.8	16.0
	3	35	7.7	3.1	20.9	7.7	**4.0**	**2.3**	6.4	2.9	14.3	5.5	53.1	14.4
**3**	1	572	8.2	4.2	23.8	8.4	2.0	1.6	4.2	2.0	17.4	7.0	55.3	12.9
	2	78	7.7	4.2	28.5	11.8	2.4	1.5	5.3	2.0	18.5	6.5	62.2	14.8
	3	24	11.9	8.9	34.1	13.2	3.2	2.4	8.4	5.6	25.4	10.2	**81.3**	**23.5**
**4**	1	723	9.3	4.7	30.8	11.2	1.6	1.2	4.5	2.7	23.4	8.6	69.2	18.0
	2	81	10.4	5.5	**38.5**	**13.2**	2.2	1.6	5.9	3.2	25.9	8.5	**82.6**	**17.7**
	3	25	11.0	5.2	**45.6**	**14.1**	2.7	2.4	9.1	4.5	32.3	13.6	**100.7**	**29.1**
**5**	1	845	10.5	4.9	37.8	11.4	1.3	1.2	4.6	3.2	27.7	9.4	81.8	18.2
	2	59	12.4	6.7	46.8	13.1	2.0	1.7	**8.1**	**4.3**	32.3	11.1	**101.1**	**21.3**
	3	31	12.2	6.0	**55.4**	**21.4**	2.3	1.5	**8.6**	**4.3**	**38.3**	**18.1**	**116.5**	**29.7**
**6**	1	804	12.1	5.8	46.0	13.7	1.2	1.1	5.3	4.2	32.4	10.6	96.8	23.3
	2	82	13.7	7.0	**60.1**	**15.7**	**2.2**	**1.7**	**8.8**	**4.7**	**41.0**	**13.4**	**125.5**	**23.7**
	3	42	12.7	5.7	**71.0**	**15.3**	**3.1**	**2.7**	**14.1**	**7.7**	**45.8**	**16.9**	**146.7**	**29.6**
**7**	1	682	13.6	6.1	54.8	15.6	1.2	1.2	5.7	4.6	38.6	11.6	113.7	24.8
	2	68	13.9	6.7	**73.0**	**21.2**	1.9	1.5	**11.3**	**6.5**	**49.4**	**17.8**	**150.1**	**32.5**
	3	35	17.5	10.3	**89.1**	**32.2**	**3.0**	**2.6**	**17.3**	**8.2**	**56.9**	**22.3**	**183.6**	**59.8**
**8**	1	657	15.2	6.9	64.9	19.2	1.1	1.0	7.2	5.9	44.4	14.1	132.7	30.0
	2	64	15.2	8.2	**86.2**	**19.9**	1.8	1.3	**14.9**	**8.4**	**54.8**	**15.8**	**172.5**	**31.1**
	3	38	17.9	7.5	**99.7**	**21.3**	**3.5**	**3.5**	**20.5**	**12.2**	**68.9**	**22.2**	**210.6**	**40.8**
**9**	1	571	17.3	8.0	74.5	21.0	1.3	1.9	8.0	6.9	51.1	15.9	152.2	33.6
	2	67	18.6	9.5	**99.7**	**21.7**	**2.1**	**2.2**	**16.5**	**10.9**	**64.9**	**19.4**	**202.3**	**33.9**
	3	34	21.2	11.5	**118.6**	**36.8**	**4.0**	**3.8**	**23.3**	**13.4**	**77.5**	**27.5**	**244.6**	**54.6**
**10**	1	535	18.1	8.6	87.0	23.9	1.2	1.3	9.9	8.3	60.0	18.4	176.2	38.9
	2	73	22.2	10.9	**117.1**	**28.0**	**2.1**	**3.0**	**17.8**	**10.9**	**71.6**	**19.1**	**230.7**	**45.6**
	3	41	**21.3**	**9.4**	**137.7**	**32.4**	**4.2**	**5.6**	**34.3**	**26.4**	**95.7**	**38.9**	**293.5**	**81.6**
**11**	1	323	19.0	8.6	97.0	26.6	1.4	2.4	11.3	10.0	63.3	18.3	192.4	44.1
	2	44	20.9	11.5	**139.8**	**33.6**	2.3	1.8	**23.6**	**14.6**	**77.6**	**22.9**	**265.9**	**47.7**
	3	24	**27.3**	**13.9**	**155.4**	**40.7**	**3.8**	**3.1**	**30.4**	**17.5**	**93.9**	**28.4**	**310.7**	**66.3**
**12**	1	288	22.4	10.7	109.5	28.5	1.4	1.2	13.6	11.0	72.2	23.5	220.3	48.4
	2	32	24.2	9.9	**153.9**	**36.5**	**3.5**	**3.7**	**26.1**	**13.7**	**89.0**	**22.3**	**296.7**	**50.1**
	3	22	27.1	13.1	**180.1**	**39.8**	**6.1**	**7.0**	**44.1**	**26.8**	**122.4**	**39.5**	**379.8**	**88.6**

Statistical significant differences comparing overweight to normal-weight and obese to normal-weight children are marked in **bold** (p<0.001).

For peak pressure measurements, an increase from the youngest to the oldest children was observed, but the differences between body mass groups were smaller than for FTI with a maximum of 1.25-fold peak pressure (total foot) in the obese children. With specific focus on the midfoot, the FTI was highest for obese (22.4 ± 19.4 N∙s), followed by over-weight (13.9 ± 10.5 N∙s) and normal-weight (7.9 ± 6.7 N∙s) children over all and in each age group (p<0.05). The obese 1 year-old children showed the smallest (1.48-fold) and the 10 year-old the highest (3.49-fold) additional midfoot loading (FTI) compared to normal-weight children (Fig 2B). The group differences for peak pressure in the midfoot ranged from 1.08-fold (1 year old) to 1.67-fold (10 year-old). The detailed data for FTI and PP for all foot regions are shown in Table 2 and Table 3. Peak pressure presents small differences for the toes, rearfoot and total foot between normal-weight, overweight and obese children. For the forefoot and midfoot, already at an age of 2 years statistical significant differences are existing (Table 3; p<0.001).

**Table 3:** The peak pressure (PP [kPa]) for the foot region: toes, forefoot (FF), medial midfoot (MMF), lateral midfoot (LMF), rearfoot (RF), total foot (TF) with mean ± SD.

age [yrs]	normal weight (1); overweight (2); obese (3)	n	Toes		FF		MMF		LMF		RF		TF	
			Mean	SD	Mean	SD	Mean	SD	Mean	SD	Mean	SD	Mean	SD
**1**	1	108	160	60	120	35	96	28	93	23	171	75	209	67
	2	29	148	42	115	25	101	39	98	33	165	73	196	63
	3	20	168	52	130	42	97	29	100	32	171	59	205	57
**2**	1	348	179	57	130	35	94	24	91	21	226	95	253	86
	2	69	170	52	131	36	101	26	97	20	213	85	240	70
	3	35	204	72	134	36	**116**	**31**	**111**	**24**	221	92	261	86
**3**	1	572	184	68	143	36	87	23	85	19	248	96	274	86
	2	78	177	68	148	38	97	27	95	21	239	95	270	90
	3	24	218	78	181	50	**108**	**24**	104	23	233	45	286	55
**4**	1	723	188	62	157	40	80	24	81	23	268	92	289	85
	2	81	206	76	167	39	90	28	89	22	252	86	284	74
	3	25	208	76	197	48	94	29	97	26	312	120	335	105
**5**	1	845	199	72	173	43	74	22	75	20	282	94	306	89
	2	59	213	83	187	37	85	23	89	19	254	79	298	75
	3	31	214	93	204	72	**95**	**33**	**96**	**30**	292	68	320	78
**6**	1	804	205	69	184	45	69	23	72	20	287	91	310	84
	2	82	238	107	**211**	**59**	**87**	**27**	**90**	**23**	280	80	323	96
	3	42	200	74	**236**	**75**	**96**	**27**	**110**	**48**	291	99	322	102
**7**	1	682	223	83	202	52	63	21	69	19	300	97	329	92
	2	68	216	76	**239**	**63**	**81**	**28**	**90**	**22**	309	101	336	93
	3	35	231	103	**253**	**62**	**100**	**26**	**110**	**21**	300	81	343	86
**8**	1	657	234	89	223	61	63	21	73	30	300	95	339	88
	2	64	237	105	**255**	**52**	**76**	**21**	**91**	**22**	304	90	351	81
	3	38	253	111	**271**	**80**	**89**	**25**	**100**	**24**	305	73	358	90
**9**	1	571	265	112	234	71	62	22	73	32	312	99	361	107
	2	67	266	125	**294**	**100**	**79**	**23**	**99**	**33**	308	83	386	114
	3	34	326	199	**306**	**76**	**97**	**30**	**111**	**28**	333	119	**428**	**168**
**10**	1	535	263	120	256	77	61	20	76	26	317	100	373	111
	2	73	298	142	**305**	**88**	**78**	**27**	**97**	**23**	331	100	414	125
	3	41	302	176	**372**	**133**	**92**	**30**	**129**	**53**	343	80	**467**	**151**
**11**	1	323	276	116	270	84	62	23	81	44	312	89	379	100
	2	44	315	165	**337**	**110**	**80**	**27**	**111**	**44**	326	82	**438**	**137**
	3	24	344	152	**351**	**76**	**98**	**32**	**117**	**32**	320	64	441	104
**12**	1	288	310	146	291	100	64	22	85	47	311	93	409	124
	2	32	322	116	**342**	**88**	**87**	**31**	**110**	**42**	339	86	436	95
	3	22	374	204	**384**	**111**	**99**	**33**	**129**	**44**	352	126	**512**	**177**

Statistical significant differences comparing overweight to normal-weight and obese to normal-weight children are marked in **bold** (p<0.001).

## Discussion

The study purposed to examine the effect of differences in body mass in children on plantar pressure distribution characteristics, calculating foot contact area, arch-index, peak pressure and force time integral in different foot regions during gait. Differences in foot loading/contact characteristics were found between body mass categories for all children and in single age groups (1–12 years). The results show higher foot contact area, reduced longitudinal foot arch, higher peak pressure and force time integral in overweight and obese children compared to normal-weight children. Thus, overweight children do not compensate additional weight by passive (foot structure) and/or active mechanisms during gait and these differences seem to be more dominant with advanced age. Additionally, the findings for this wide age range shows for the first time clear evidence for age dependent differences and loading patterns that have to be discussed in the context of foot development in children.

The higher foot contact area in overweight and obese children compared to normal-weight counterparts, is in agreement with previous results [12,19]. It needs to be emphasized that this finding was observed already in the youngest children. The arch index rises with additional body mass and is getting statistically significant with an age older than 4 years. Similar data was only shown in small specific age ranges in children [19,22]. Since the arch index cannot clearly distinguish between the different causes of higher midfoot contact area regarding flat or fat feet, the results have to be questioned for functional relevance. But, for a static situation Villaroya et al. (2008) found a relationship of high midfoot loading/contact area and lower longitudinal arch, validated by radiographic assessment [31]. This supports the validity of the arch index to identify the characteristics of the longitudinal foot arch. Nevertheless, the judgment of a higher arch index as positive, negative or neutral is under debate. Tudor et al. (2009) could not find any disadvantage for children with flat-footedness concerning performance, also a higher risk of injury amongst athletes with pes planus was not reported [32]. Furthermore, is not clear whether children with high arch index will suffer from foot pathologies or pain later in life [33–35]. Finally, it has to be questioned if a categorization of foot types (flat, normal, high longitudinal arch) based on arch index is reasonable, if the consequences are not yet discussed [30,36].

The foot loading, quantified by the FTI and PP, was higher for overweight and obese children. This was most distinct for the midfoot (FTI and PP) despite the fact of a larger foot contact area in this foot section possibly reducing midfoot PP in children with higher BMI. This is in contrast to some studies showing only weak to moderate correlations between PP and body mass in children and adolescents [22]. Other studies reported similar foot loading changes in obese compared to normal-weight children or adults [3,8,12,37]. Questioning the functional relevance, the up to 3.5-fold higher loading of the midfoot in obese children compared to normal-weight children appears as an alarming value. Obese children seem not to be capable to compensate the extra body mass leading to an equally plantar load distribution across all foot regions compared to non-obese children. In contrast, they even show a disproportional elevation of midfoot loading additionally increasing with age. Considering all age groups and foot regions, characteristic loading patterns for overweight and obese children are visible as marked in Tables 2 and 3. The overall loading of the foot represented by the FTI is showing a different pattern then the peak pressure (PP), a measure of the maximum isolated loading. Overweight and obesity especially impacts the main weight bearing regions of the foot (rearfoot, forefoot and total foot) followed by the midfoot in the children aged 5years and older. In contrast the PP is most effected in the midfoot and forefoot and lesser for the rearfoot. This could be interpreted as an adaptation strategy to compensate additional body weight in the obese children [20].

Sports and physical activity could be hypothesized as reasonable preventive strategies to improve active compensation of body mass and loading on the (mid-) foot. It is often hypothesized that higher foot loading and therefore higher stress on the foot structures is connected to discomfort or pain [3]. Taking this into account, overweight and obesity has to be seen critically for foot health already in early childhood beginning at an age of 1 year. It could also be speculated that higher foot stress and possible discomfort could serve as a factor negatively influencing activity level in these children starting a vicious circle.

In the context of general foot and gait development, foot characteristics of young children seem to be affected by additional body mass. This is valid to a higher extent in older compared to younger children. In line with previous results, the arch index for normal, overweight and obese children shows a reduction for 1–5 year old children with a leveling off starting at the age of 5 to 6 years [16].

The strength of the study is the large cohort of children aged 1–12 years. However, there are some limitations that need to be considered. Noticeable large standard deviations for the obese groups in all measures should be seen in context of a possibly higher interindividual variability in obese and the small sample size in these groups compared to the overweight and normal-weight groups. Therefore, high standard deviations in the age and weight groups have to be considered for interpretation. This variability will exacerbate the development and determination of cut off point for critical foot loading patterns. Self-chosen gait velocity in the present study showed no differences between BMI categories. This might in part be attributed to the restricted length of the walkway. However, the use of the two-step protocol and a similar walking velocity between BMI categories ensure that the impact on plantar pressure measurements may be considered negligible [16,28]. Gait asymmetries between left and right feet could not be presented due to the chosen measurement protocol of only the right side. This was defined to keep the compliance and data quality for repetitive gait trials in children high. Furthermore, the calculated arch index is an indirect measure of the foot longitudinal arch height leading to restraints in interpretation.

Due to field measurements body mass was assessed while wearing all day clothes. No correction was applied to avoid additional bias in short to tall children, which means that data interpretation is not valid on an individual level.

## Conclusion

In summary, the study presents detailed data for substantial differences in dynamic foot characteristics for normal-weight, overweight and obese children for each age between 1 to 12 years. Additional body mass showed a reduction of the foot arch index and higher overall loading impacting especially the midfoot area disproportionately. Moreover, plantar pressure evaluation from onset of unsupported walking to mature similar gait in different foot regions display specific patterns for overweight and obese children. Overweight and obesity are not compensated by the musculoskeletal system and already affect the one and two year old children´s feet and becomes progressively pronounced with increasing age. To avoid excessive foot loading with potential risk of discomfort or pain in childhood, prevention strategies should be developed and validated for children with a high body mass index. The presented plantar pressure values could additionally serve as reference data to identify suspicious foot loading patterns in children.
